# Neck circumference in Latin America and the Caribbean: A systematic review and meta-analysis

**DOI:** 10.12688/wellcomeopenres.16560.1

**Published:** 2021-01-26

**Authors:** Patricia A. Espinoza López, Kelly Jéssica Fernández Landeo, Rodrigo Ricardo Pérez Silva Mercado, Jesús José Quiñones Ardela, Rodrigo M. Carrillo-Larco

**Affiliations:** 1 School of Medicine “Alberto Hurtado", Universidad Peruana Cayetano Heredia, Lima, Peru; 2 Department of Epidemiology and Biostatistics London, School of Public Health, Imperial College London, London, UK; 3 CRONICAS Centre of Excellence in Chronic Diseases, Universidad Peruana Cayetano Heredia, Lima, Peru

**Keywords:** Anthropometrics, cardio-metabolic risk factor, obesity

## Abstract

**Background: **High neck circumference (NC) is associated with high burden diseases in Latin American and the Caribbean (LAC). NC complements established anthropometric measurements for early identification of cardio-metabolic and other illnesses. However, evidence about NC has not been systematically studied in LAC. We aimed to estimate the mean NC and the prevalence of high NC in LAC.

**Methods: **We conducted a systematic review in MEDLINE, Embase, Global Health and LILACS. Search results were screened and studied by two reviewers independently. To assess risk of bias of individual studies, we used the Hoy *et al.* scale and the Newcastle-Ottawa scale. We conducted a random-effects meta-analysis.

**Results: **In total, 182 abstracts were screened, 96 manuscripts were reviewed and 85 studies (n= 51,978) were summarized. From all the summarized studies, 14 were conducted in a sample of the general population, 23 were conducted with captive populations and 49 studies were conducted with patients. The pooled mean NC in the general population was 35.69 cm (95% IC: 34.85cm-36.53cm; I²: 99.6%). In our patient populations, the pooled mean NC in the obesity group was 42.56cm (95% CI 41.70cm-43.42cm; I²: 92.40%). Across all studied populations, there were several definitions of high NC; thus, prevalence estimates were not comparable. The prevalence of high NC ranged between 37.00% and 57.69% in the general population. The methodology to measure NC was not consistently reported.

**Conclusions: **Mean NC in LAC appears to be in the range of estimates from other world regions. Inconsistent methods and definitions hamper cross-country comparisons and time trend analyses. There is a need for consistent and comparable definitions of NC so that it can be incorporated as a standard anthropometric indicator in surveys and epidemiological studies.

## Abbreviations

Body Mass Index: BMI, Latin American and the Caribbean: LAC, Neck Circumference: NC, Obstructive Sleep Apnea - Hypopnea Syndrome: OSAHS, Waist Circumference: WC

## Introduction

Anthropometric indicators have an important role in public health because they are risk factors or diagnostic criteria for some highly prevalent non-communicable diseases (e.g., cancers and cardio-metabolic diseases) ^1– 4^. Weight, height, body mass index (BMI) and waist circumference (WC) have been broadly studied in terms of prevalence and time trends ^4, 5^, and their long-term association with health outcomes has been studied by large cohorts in many world regions ^2, 3^. This evidence for neck circumference (NC) lacks globally and in Latin American and the Caribbean (LAC), where novel and inexpensive anthropometric indicators could contribute to the prevention and early identification of non-communicable diseases ^6– 8^.

Unlike BMI, there have been no efforts to summarize mean NC and prevalence estimates of high NC in LAC. This evidence could provide a baseline parameter of this anthropometric indicator to inform future research and surveillance plans, while also characterizing population groups in terms of their NC profile. In addition, a critical appraisal of the available evidence about NC in LAC lacks, thus research gaps, needs and methodological issues have not been identified to improve the formulation of future research. With evidence that NC appears to be a risk factor for many diseases (e.g. cardio-metabolic diseases and obstructive sleep apnea), in a similar magnitude as other anthropometric indicators ^7– 10^, it becomes relevant to understand the current status of NC so that it could be incorporated in population-based surveys or epidemiological studies.

To summarize the evidence about NC in LAC, to provide pooled estimates of mean levels and prevalences, and to highlight research needs and methodological caveats, we conducted a systematic review and meta-analysis of the scientific evidence about NC in LAC populations.

## Methods

### Protocol and registration

This is a systematic review of the scientific literature with meta-analysis of summary data. The methodology and reporting followed the PRISMA guideline (see reporting guidelines) ^11^. The protocol was prepared before conducting the review and is available online ^12^.

### Eligibility criteria

This review included original studies with the following populations: LAC adults either from the general population, captive/closed populations (e.g., workers) or patients from any healthcare facility. We excluded patients who had conditions that could have biased the NC measurement (e.g., cervical masses, thyroid diseases, cervical fractures or congenital anomalies). We excluded studies with LAC immigrants in countries outside the LAC region. Studies should have reported that NC was measured, regardless of the methodology; in other words, if NC was not directly measured (i.e., NC was self-reported), this study was excluded. We excluded case reports, case series, letters, editorials, narrative reviews, clinical trials and systematic reviews.

### Information sources and search

We conducted the search in MEDLINE, Embase and Global Health, these three were searched through OVID; we also searched LILACS, a LAC specific search engine. The search was conducted on 27 September 2020. The complete search strategy is available as extended data ^13^.

### Study selection

First, two authors (KFL, RPSM / PEL, JQA) independently screened the titles and abstracts of the search results. Second, the same reviewers independently studied the full text of the selected articles. Likewise, the full text of the selected articles was analyzed to ensure that multiple publications of the same study were included once only (e.g., national survey with multiple publications). Discrepancies between reviewers were resolved by consensus between them or by discussions with a third reviewer (RMC-L). If the information reported in the original article was not enough to assess the eligibility criteria, we tried to contact the corresponding author of these studies. Those articles which corresponding authors did not answer to our communications after two weeks were excluded from this review.

### Data collection process

Two authors (KFL, RPSM / PEL, JQA) independently extracted the information from the included articles using a standard form for each of the population groups herein studied (general population, captive population and patients). Any differences between the two reviewers were resolved by consensus between them or by discussions with a third reviewer (RMC-L). The extraction form we used was developed before data collection and was not modified during the extraction process.

### Data items

The following information was extracted from all articles: title, first author, country, publication year, year of data collection, study design, sample size, mean age and age range of the study population, men proportion, instrument and method to measure NC, cut-off point of high NC overall and by gender, prevalence of high NC overall and by gender, and mean NC overall and by gender. From articles with a sample of the general population, we also extracted information on whether it was a national sample. From articles with a captive population, we also extracted the origin of the population (e.g., students or elderly in nursing homes). From articles with patients we recorded the underlying disease. Additionally, the following information was extracted from the case-control studies: proportion of cases and controls, mean NC for cases and controls, and prevalence of high NC for cases and controls.

### Risk of bias of individual studies

Two authors (KFL, RPSM / PEL, JQA) independently assessed the risk of bias of the articles using the risk of bias tool for prevalence studies by Hoy *et al.* ^14^; we used the Newcastle-Ottawa scale for the case-control and cohort studies ^15^. Discrepancies between the two reviewers were solved by discussion with a third reviewer (RMC-L). Items that did not apply (e.g., acceptable case definition for prevalence studies) to our selected reports were not assessed.

### Synthesis of results

We conducted a quantitative synthesis (meta-analysis) of mean NC only, because evidence from prevalence estimates was largely heterogeneous (e.g., different definitions) and scarcer than for mean estimates. We decided to conduct the meta-analysis when there were at least three individual estimates ^12^. We only conducted the meta-analysis for overall mean estimates (i.e., not sex-stratified). Using the mean estimates along with the corresponding standard errors computed from the confident intervals [standard error = (upper limit - lower limit)/3.92], we conducted a random-effects meta-analysis in STATA v16.1 (College Station, Texas 77845 USA); we used the *metan* function with the *randomi* option for a random-effects model following the DerSimonian & Laird method.

### Ethics

This is a systematic review of the scientific literature in which human subjects were not directly studied. We did not request approval by an Ethics Committee. All authors had access to the collated data and are collectively responsible for the accuracy of results and conclusions. All authors approved the submitted version. The funder had no role in the study design, analyses, interpretation or conclusions.

## Results

### Study selection

**Figure 1.**
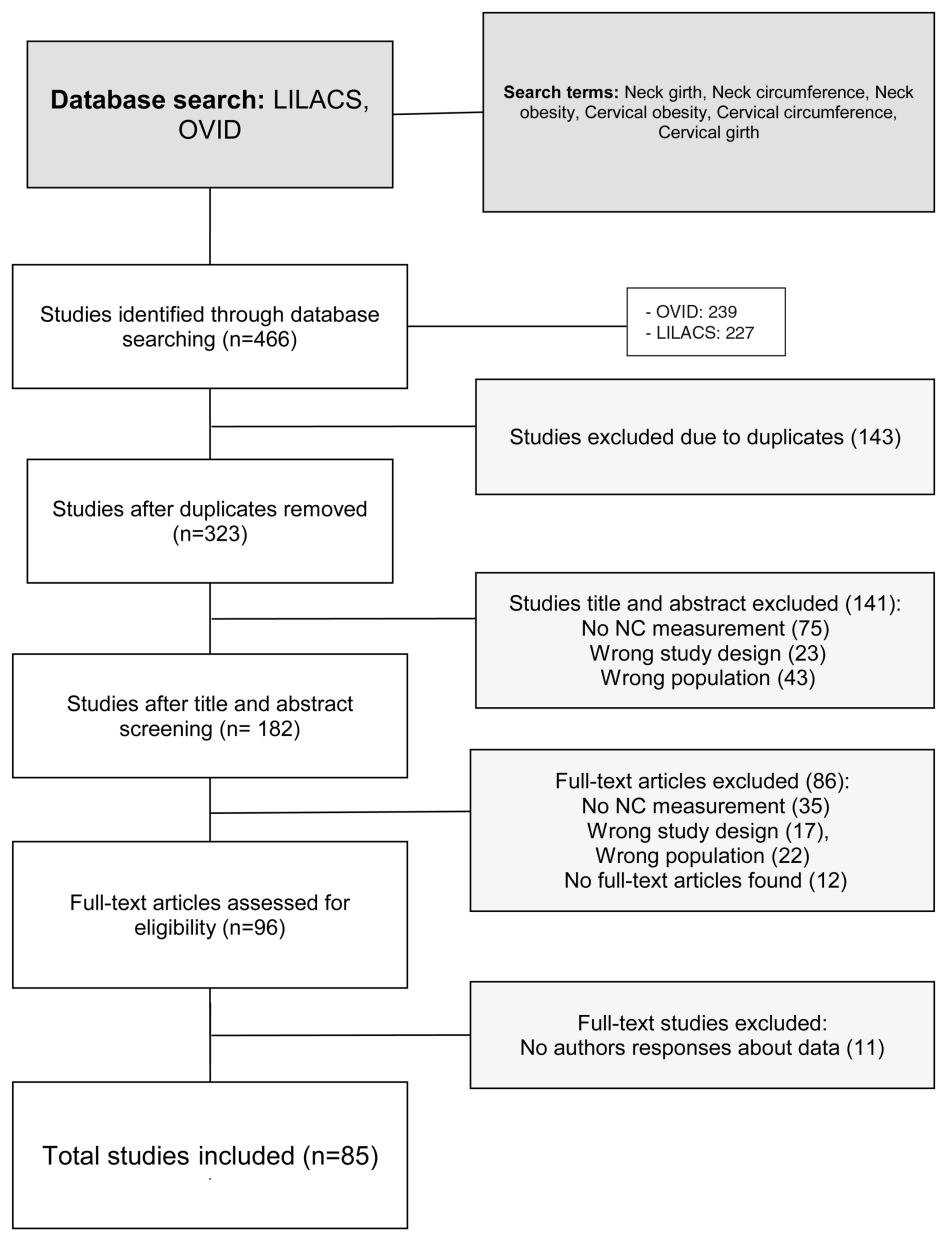
Selection flow chart.

The article search yielded 323 results; of these, 182 titles and abstracts were screened and then, 96 manuscripts were studied. We finally included 85 (n=51,978) studies ( Figure 1) ^16– 100^.

### Neck circumference in the general population

Of the total selected articles, 14 studies ^16– 29^ were conducted in a sample of the general population (one study also contributed to the captive population group ^29^). The 14 studies followed a cross-sectional design. Most of them were from Brazil (10) ^16– 18, 20– 24, 26, 28^ while the rest were from Argentina ^25^, Chile ^29^, Colombia ^27^ and Venezuela ^19^. The age of the study population was ≥18 years old, except in one study which was ≥15 years ^29^. The total sample was 24,401, with a mean age of 39.73 years. The instrument or methodology to measure NC was detailed in 11 studies ( Table 1) ^17– 19, 21– 27, 29^.

**Table 1.** Synthesis of population-based studies.

Study Information												Neck Circumference (cm)						Contribution to Meta- analysis
First Author	Country	Data Year	Study Design	Sample	Mean Age	Age Range	Men Proportion	Instrument	Measured How	Cut Point for Men (cm)	Cut Point for Women (cm)	Mean Overall	Mean Men	Mean Women	Prevalence Elevated Overall	Prevalence Elevated Men	Prevalence Elevated Women	
Moraes, W. *et al.* ^16^	Brazil		Cross - sectional	1042	42.20	20 – 80	44.91					36.20	39.40	33.60				Yes
Neves, T. *et al.* ^17^	Brazil	2010	Cross - sectional	387	71.00	65 – 93	36.43	Measuring tape	The mean height of the neck.			35.46	38.00	34.00				No
Ribeiro, L. *et al.* ^18^	Brazil	2018	Cross - sectional	130		≥18	32.31	Measuring tape	The cricothyroid cartilage height level was used as a reference for the measurement. For men, the NC was measured just below the cartilage because of the greater prominence of this region.	37.00	34.00				57.69			No
Méndez- Pérez, B. *et al.* ^19^	Venezuela	2015	Cross - sectional	1132		15 – 65	48.76	Measuring tape		39.00	35.00	36.17	38.17	34.27	46.75	48.70	44.80	Yes
Leite, J. *et al.* ^20^	Brazil		Cross - sectional	2027			40.90					35.62						Yes
Volaco, A. *et al.* ^21^	Brazil	2014	Cross - sectional	950	47.40	≥18	34.64	Measuring tape	At the middle high of the neck, bellow the laryngeal prominence (Adams’s apple), around the neck, parallel to the floor.	39.50	34.50	35.50	38.20	34.10				Yes
Stabe, C. *et al.* ^22^	Brazil	2004	Cross - sectional	1053	39.40	18 – 60	28.58	Measuring tape	Below cricothyroid cartilage.	40.00	36.10	36.98	39.70	35.90				Yes
Zanuncio, V. *et al.* ^23^	Brazil	2013	Cross - sectional	948	38.34	20 – 59	44.94	Measuring tape	Below laryngeal prominence.	39.50	33.30	35.59	38.63	33.11				Yes
Chaves, T. *et al.* ^24^	Brazil	2011	Cross - sectional	365	43.90	18 – 65	43.83	Measuring tape		40.50	34.20	33.60						Yes
Alfie, J. *et al.* ^25^	Argentina	2008	Cross - sectional	3987	43.80	≥18	48.40	Measuring tape	Base of the neck.	41.00	35.00				0.37			No
Soares, M. *et al.* ^26^	Brazil		Cross - sectional	993	41.80	20 – 80	46.12		At the level of the cricothyroid membrane.	43.00	38.00	36.33	39.40	33.70				Yes
Ruiz, A. *et al.* ^27^	Colombia	2013	Cross - sectional	6074	40.10	≥18	46.20	Measuring tape	Above laryngeal prominence, perpendicular to the neck axis.	43.18	40.64	35.96	38.78	34.87				Yes
Polesel, D. *et al.* ^28^	Brazil		Cross - sectional	407	34.80	20 – 80	0.00					33.75	0.00	33.30				Yes
Mora, R. *et al.* ^29^	Chile	2016	Cross - sectional	4906	31.50	≥15	40.00	Measuring tape	At the level of most prominence of the cricoid cartilage (Adam’s apple).	41.00	35.00	36.90	39.10	34.70				Yes

Mean NC was available from 12 articles (n=20,284) ^16, 17, 19– 24, 26– 29^, though the pooled mean NC was based on 11 estimates ^16, 19– 24, 26– 29^: the overall pooled mean NC was 35.69cm (95% IC: 34.85cm-36.53cm; I²: 99.6%) ( Table 1). The minimum and maximum mean NC in men were 38.17cm and 39.70cm, respectively; while these numbers in women were 33.11cm and 35.90cm, respectively ( Table 1). The prevalence of high NC was available from 3 studies ^18, 19, 25^, all of which used different thresholds for men and women; for men, the cut-off points ranged between 37cm and 41cm, while for women the range was between 34cm and 35cm. Based on these definitions, the prevalence of high NC in the general population went from 37.00% to 57.69% ( Table 1). One study ^19^ reported the prevalence of high NC stratified by sex; for men, high NC was defined at >39cm while for women this cut-off point was >35cm, yielding a prevalence of 48.70% in men and 44.80% in women ( Table 1).

### Neck circumference in captive populations

Of the total selected articles, 23 reports ^30– 51^ included captive populations and 1 ^38^ of these reports provided 2 estimates (i.e., two different populations). Studies followed a cross-sectional, case-control and cohort design. Most of the articles were from Brazil (19) ^30– 35, 37– 45, 48– 51^ while the others were from Chile (3) ^29, 36, 46^ and Peru (1) ^47^. The instrument and methodology to measure NC was reported in 19 studies ( Table 2) ^29, 31– 34, 36, 37, 39– 46, 48, 49– 51^.

**Table 2.** Synthesis of reports in selected populations.

Study Information													Neck Circunference (cm)					
First Author	Country	Data Year	Study Design	Population Type	Sample	Mean Age	Age Range	Men Proportion	Instrument	Measured How	Cut Point Men	Cut Point Women	Mean Overall	Mean Men	Mean Women	Prevalence Elevated Overall	Prevalence Elevated Men	Prevalence Elevated Women
Tavares, C. *et al.*. ^30^	Brazil		Cross- sectional	Healthcare professionals	159	43.20		21.38					34.74	39.30	33.50			
Dantas, E. *et al.* ^31^	Brazil		Cross- sectional	University students	406	20.95		33.25	Measuring tape	Above the thyroid cartilage prominence.	39.60	36.10	33.66	36.95	32.02			
De Siquiera, K. *et al.* ^32^	Brazil	2011	Cross- sectional	University students	691		18 – 58	37.80	Measuring tape	Below the superior border of the prominence of the larynx.	39.00	35.00	37.10					
De Alexandria, F. *et al.* ^33^	Brazil	2011	Cross- sectional	Female nurse	71.0	42.00	18 – 69	0.00	Measuring tape	Midpoint of the neck height.		34.00	33.00		33.00			
Alves, H. *et al.* ^34^	Brazil	2010	Cross- sectional	University students	702	21.50	≥18	37.40	Measuring tape	The measuring tape was positioned just below the top edge of the laryngeal prominence.	39.00	35.00	33.70					
Santosa, D. *et al.* ^35^	Brazil	2008	Cross- sectional	Professional urban bus drivers	404	38.20		100.00					39.10					
Pedreros, A. *et al.* ^36^	Chile		Cross- sectional	Miners	221	38.00		100.00	Measuring tape		38.00		39.60	39.60				
Haueisen, M. *et al.* ^37^	Brazil	2009	Cross- sectional	Active or retired civil servants of universities or research institutions /Brazilian Longitudinal Study of Adult Health (ELSA–Brasil)	11221	51.50	35 – 74	48.50	Measuring tape	Right above the cricoid cartilage and perpendicular to the long axis of the neck, with the participant in a sitting position.			36.62	39.50	33.90			
Genta, P. *et al.* ^38^	Brazil	2004	Case - Control	Japanese descendants in São Paulo, Brazil	54	53.30	≥18	100.00					40.00					
Genta, P. *et al.* ^38^	Brazil	2004	Case - Control	White males in São Paulo, Brazil	466	50.60	≥18	100.00					42.00					
Nogueira, M. *et al.* ^39^	Brazil	2012	Cross- sectional	Elderly at a health center	411	70.00		26.00	Measuring tape	The base of the neck, below the laryngeal prominence			35.11	39.70	33.50			
Barbosa, P. *et al.* ^40^	Brazil	2015	Cross- Sectional	Adults attended at a health center	126	36.20	18 – 59	19.00	Measuring tape	Midpoint of the cervical spine to the anterior half of the neck. In men with laryngeal prominence.			34.00					
Frizon, V. *et al.* ^41^	Brazil	2013	Cross-Sectional	Patients waiting for medical, dental, psychological, or nutritional consultation	155	38.00	19 – 60	29.00	Measuring tape	At the base of the neck, at the height of the cricothyroid cartilage. In men with prominence, NC was measured below prominence.	37.00	34.00	36.10	40.10	34.50	54.80	86.70	41.80
Coelho, H. *et al.* ^42^	Brazil	2015	Cross- sectional	Elderly at a health center	435	68.28	≥18	14.80	Measuring tape	Just above the cricoid cartilage and perpendicular to the long axis of the neck	40.5	35.7	36.94	40.20	36.38	54.25	50.79	54.99
Da Silva, A. *et al.* ^43^	Brazil		Cross- Sectional	General outpatient nutrition clinic of a public university hospital specialized in cardiology	129	55.60	≥35	26.40	Measuring tape	long the axis of the neck at the midpoint of the cervical spine to the midanterior of the neck.	37.00	34.00	36.60	40.30	35.70			
Closs, V. *et al.* ^44^	Brazil	2012	Cross- sectional	Elderly at a health center	583	68.50	60–103	36.37		Over laringeal prominence.			36.86	39.19	35.53			
Petreça, D. *et al.* ^45^	Brazil	2016	Cross- sectional	Elderly at a health center	170	69.50	≥60	0.00	measuring tape	Above thyroid cartilage.			34.60		34.60			
Pizarro- Montaner, C. *et al.* ^46^	Chile		Cohort	Miners	111	34.70	25–44	100.00		Below laringeal prominence.	37.00		39.95	39.95				
Peralta, L. *et al.* ^47^	Peru	2011	Cross- sectional	Medical students	46	19.60	18–23	50.00					35.40	37.30	33.50			
Dos reis, E. *et al.* ^48^	Brazil		Cross- sectional	Elderly caregivers	34	43.68	≥18	100.00	measuring tape		34.00		35.87	35.87		62.00	62.00	
Ferreira, A. *et al.* ^49^	Brazil	2016	Cross- sectional	Mura ethnia	455	42.10	18–81	42.20	measuring tape	Horizontal plane of Frankfort.	37.00	34.00	36.68					
Ramires, A.R. *et al.* ^50^	Brazil	2011	Cross- sectional	Sedentary women	60	33.90	≥18	0.00	measuring tape	Below laryngeal prominence.		35.00			33.78			55.00
Mora, R. *et al.* ^29^	Chile	2016	Cross- sectional	Outdoor Gym Users	1023	31.50	≥15	70.97	measuring tape	Cricoid cartilage prominence.	41.00	35.00	38.22	39.30	35.60			
Sgariboldi, D. *et al.* ^51^	Brazil		Cross- sectional	Sedentary women	100	44.59	25–75	0.00	measuring tape	Cricotiroid cartilage level.			35.84		35.84			

Of the 23 articles in this group, 22 articles (n=18,173) reported the mean NC. Additionally, 14 studies ^29, 31– 36, 41– 43, 46, 48– 50^ established different cut-off points for high NC for men and women, with a minimum and maximum value for men of 37cm and 42cm, respectively; while the values for women were 34 cm and 36.10 cm, respectively ( Table 2).

From all the captive population articles, 4 of these included university students ^31, 32, 34, 47^ and also reported the mean NC ( Table 2). The minimum and maximum mean NC in this captive population were 33.66cm and 37.10cm, respectively; in men were 36.95cm and 37.30cm, respectively; and in women the minimum and maximum values were 32.02cm and 33.50cm, respectively ( Table 2).

Of all the articles with captive populations, 4 of these included elderly people ^39, 42, 44, 45^ and also reported the mean NC. The minimum and maximum mean NC in this captive population were 34.60cm and 36.94cm, respectively; in men were 39.19cm and 40.20cm, respectively; and in women the minimum and maximum values were 33.50cm and 36.38cm, respectively ( Table 2). 

The prevalence of high NC was available from 3 studies ^41, 42, 50^, all of which used different thresholds for men and women; for men, the cut-off points ranged between 34cm and 40.5cm, while for women the range was between 34cm and 35.7cm ( Table 2). Based on these definitions, the prevalence of high NC in the overall sample of captive populations went from 54.25% to 62%; while for men and women it went from 50.79% to 86.70%, and from 41.80% to 54.99%, respectively ( Table 2). 

### Neck circumference in patients

Of the total selected studies, 49 reports ^52– 100^ were conducted with patients. Studies followed a cross-sectional, case-control and cohort design. Most of the articles were from Brazil (35) ^52– 57, 60, 61, 63– 65, 70, 71, 73– 86, 88, 91– 93, 95– 97, 99^ while the others were from Chile (5) ^58, 68, 87, 89, 94^, Mexico (4) ^66, 67, 90, 100^, Peru (3) ^62, 69, 72^ and Argentina (2) ^59, 98^. The most frequently studied patients were those with Obstructive Sleep Apnea - Hypopnea Syndrome (OSAHS) (22) ^53, 54, 58– 62, 64– 69, 74, 75, 79, 81, 86, 87, 90, 94, 97, 98^ and obesity (13) ^52, 71, 73, 80, 82– 85, 91, 92, 95, 99, 100^; other diseases included HIV/AIDS, sleep disorders, bronchiectasis, depression, stroke, epilepsy, hepatic and cardiovascular pathologies. The instrument or methodology to measure NC was reported in 29 studies ^54– 57, 60, 63, 65– 67, 69– 71, 73, 77, 80– 82, 84, 86, 88– 93, 95, 97, 99, 100^. We found 1 report ^53^, which contributed with 2 estimates (e.g., one report provided more than one set of estimates).

Of the 49 studies in this group, 44 studies (n=8,059) ^52– 57, 59– 61, 63, 65, 66, 68– 73, 75– 100^ reported the mean NC. The overall pooled mean NC in patients with OSAHS (19 estimates; n=4,141) ^53, 54, 58, 60, 61, 65, 68, 69, 75, 79, 81, 86, 87, 90, 97, 98^ was 41.09cm (95% CI 40.42cm-41.77cm; I²: 94.80%), and for those with obesity (13 estimates; n=1,952) ^52, 71, 73, 80, 82– 85, 91, 92, 95, 99, 100^ the overall pooled mean NC was 42.56cm (95% CI 41.70cm-43.42cm; I²: 92.40%) ( Table 3). In studies that included patients with OSAHS, the minimum and maximum mean NC were 37.40cm and 44.50cm, respectively ( Table 3). Studies with obese patients, the minimum and maximum mean NC were 37.01cm and 44.41cm, respectively ( Table 3).

**Table 3.** Synthesis of hospital-based studies.

Study Information													Neck Circunference (cm)									
First Author	Country	Study Design	Disease	Sample	Mean Age	Men Proportion	Case Proportion	Instrument	Measured How	Cut Point (cm)	Cut Point for Men (cm)	Cut Point Women (cm)	Mean Overall	Mean Men	Mean Women	Mean Cases	Mean Controls	Prevalence Elevated Overall	Prevalence Elevated Men	Prevalence Elevated Women	Prevalence Elevated Cases	Prevalence Elevated Controls
De Paiva, R, *et al*. ^52^	Brazil	Case - Control	Obesity	45	46.50	31.10	68.90						43.40			44.60	39.50					
Zonato, A, *et al.* ^53^	Brazil	Cross - sectional	OSAHS / Public Clinic	307	50.00	67.00	33.00						41.00			41.00						
Zonato, A, *et al.* ^53^	Brazil	Cross - sectional	OSAHS / Private Clinic	317	48.00	87.00	13.00						43.00			43.00						
Sutherland, K, *et al.* ^54^	Brazil	Cross - sectional	OSAHS	137	48.10	69.50	30.50	Measuring tape	Below the laryngeal prominence with tape measure perpendicular to the long axis of the neck.				41.80			41.80						
Pinto, J, *et al.* ^55^	Brazil	Cross - sectional	Sleep disorders	82	43.76	80.50	19.50		Along a horizontal line across the midline of the thyroid cartilage.				39.87									
Oliveira, N, *et al.* ^56^	Brazil	Cross - sectional	HIV/AIDS	35	43.90	51.40	48.60	Measuring tape			37.00	34.00	35.20	36.90	33.40							
Musman, S, *et al.* ^57^	Brazil	Cross - sectional	Sleep disorders	323	44.60	59.13	40.87	Measuring tape	The level of the cricoarytenoid joint.				40.00	42.00	37.00	42.00	38.00					
Salas, C, *et al.* ^58^	Chile	Cross - sectional	OSAHS	1044	53.20	76.00	24.00									42.10	38.30					
Saban, M, *et al.* ^59^	Argentina	Cross - sectional	OSAHS	302	56.00	55.96	44.04						41.00			42.00	37.00					
Moura, P, *et al.* ^60^	Brazil	Cohort	OSAHS	102	46.75	55.90	44.10		At the level of the cricothyroid membrane.				38.47	40.86	35.44	38.47						
Souza, F, *et al.* ^61^	Brazil	Cross - sectional	OSAHS	10	48.90	40.00	60.00						40.35			40.35						
De Castro, J, *et al.* ^62^	Peru	Case - Control	OSAHS	95	48.90											43.32						
Boemeke, L, *et al.* ^63^	Brazil	Cross - Sectional	Non-alcoholic fatty liver disease	82	41.70	33.00	67.00	Measuring tape	Below the prominence of the larynx and perpendicular to the long axis of the neck, with the tape positioned at the same height at the front and at the back of the neck. The individual was asked to remain in an upright position, with proper posture and looking forward.		42.00	36.00	41.70	46.50	39.40			86.60				
Hiray, M, *et al.* ^64^	Brazil	Cross - Sectional	OSAHS	48	34.00	79.20	20.80			40.00						42.00	38.00				100.00	64.10
Borges, P, *et al.* ^65^	Brazil	Cross - Sectional	OSAHS	93	46.70	58.10	41.90		At the level of the cricothyroid cartilage.				38.56	40.91	35.31	38.56						
Saldaña,R, *et al.* ^66^	Mexico	Cross - Sectional	OSAHS	10	44.90	60.00	40.00		At the level of the cricothyroid membrane.				41.80									
Castorena- Maldonado, A, *et al.* ^67^	Mexico	Case - Control	OSAHS	61	35.50	62.30	37.70	Measuring tape	At the level of the cricothyroid membrane.							39.80	34.50					
Jorquera, A, *et al.* ^68^	Chile	Cross - Sectional	OSAHS	457	52.27	80.00	20.00						39.20			39.20						
De Castro, J, *et al.* ^69^	Peru	Cohort	OSAHS	408	49.40	91.00	9.00		Technique proposed by Lohman.				41.55			41.55						
Rodrigues, A, *et al.* ^70^	Brazil	Cross - sectional	Depression	79	XXX	24.10	75.90	Measuring tape	The participant standing upright and the measurement was taken at mid-neck height.		37.00	34.00	33.41					30.40				
Aguiar, I, *et al.* ^71^	Brazil	Cross- Sectional	Obesity	38	42.00	13.16	86.84		Horizontally at the level of the cricoid cartilage.		43.00	41.00	42.60	44.48	42.32	42.60						
Chávez- Gonzáles, C, *et al.* ^72^	Peru	Cross - Sectional	Snore	230	49.76	56.50	43.50						40.60									
De Menezes, R, *et al.* ^73^	Brazil	Cross - Sectional	Obesity	1089	38.10	28.30	71.70		At the level of the cricoid membrane with the patients in the supine position.	42.00			42.30	47.50	40.30	42.30						
Cunha, F, *et al.* ^74^	Brazil	Case - Control	OSAHS	37	33.94											41.90	35.50					
Prescinotto, R, *et al.* ^75^	Brazil	Cohort	OSAHS	28	48.80	32.10	67.90						38.30			38.30						
Faria, J, *et al.* ^76^	Brazil	Cross - sectional	Bronchiectasis	21	51.60	42.90	57.10						38.81									
Schommer, V, *et al.* ^77^	Brazil	Cross - sectional	Heart failure	123	61.90	60.20	39.80	Measuring tape	Midpoint of the neck.				37.86									
Amaro, T, *et al.* ^78^	Brazil	Cross - sectional	Acute myocardial infarction	34	61	58.8	41.2				37	34	37.5					75.20	95.00	50.00		
Nerbass, F, *et al.* ^79^	Brazil	Cross - sectional	OSAHS	90	46	51	49				43	41	37.4			39	36.4					
Lucas, E, *et al.* ^80^	Brazil	Cross - sectional	Obesity	147	40.71	34.69	65.31	Measuring tape	Individuals seated on a chair, the head in a neutral position, looking straight ahead. Around the neck, Over thyroid cartilage.		42	36	41.3	45.4	39.1	44.9	34.2					
Freire, L, *et al.* ^81^	Brazil	Cross - sectional	OSAHS	50	57.52	42	58	Measuring tape	Participants stood up straight, with their heads positioned in the horizontal plane of Frankfort. Below the laryngeal prominence and applied perpendicularly along the neck axis.				39.14			39.38	35.33					
Sgariboldi, D, *et al.* ^82^	Brazil	Cross - sectional	Obesity	156	44.46	0	100	Measuring tape	Cricoid cartilage.				37.01		37.01	41.25	32.55					
Martinho, F, *et al.* ^83^	Brazil	Cross - sectional	Obesity	45	46.5	31.11	68.88						43.4			43.46	39.5					
Correa, M, *et al.* ^84^	Brazil	Cross - sectional	Obesity	81	42	27.16	62.84	Measuring tape	Cricoid cartilage.				38.8			38.8	38					
Magalhaes, E, *et al.* ^85^	Brazil	Case - Control	Obesity	88	49.2	21.6	78.4						38.5			40.7	36.4					
Menezes, D, *et al.* ^86^	Brazil	Cross - sectional	OSAHS	456	43.7	63.8	36.2	Measuring tape	Cricothyroid membrane.	40			40.8			41.6	38.1	51.8				
Saldias, P, *et al.* ^87^	Chile	Case - Control	OSAHS	328	52	82	18			41	43	40	42.72			44	41.3	57.92				
Mendes, C, *et al.* ^88^	Brazil	Cohort	Stroke	89	64.39	64	36		Below larynx.		43	38	40.33			40.33	40.05	39.32	34.2	65.8		
Miño, F, *et al.* ^89^	Chile	Cross - sectional	Cardiovascular disease	112	55	34.51	65.49	Measuring tape					39.31			37.4	38.93					
Garcia, J, *et al.* ^90^	Mexico	Cohort	OSAHS	146	47.6	67.1	32.9		Cricothyroid membrane.	40			41.04			41.04		61.64				
Lima, J, *et al.* ^91^	Brazil	Cohort	Obesity	42	42.5	40.5	59.5		Cricoid cartilage.				42.2	47.2	40.5	42.2						
Padilha, L, *et al.* ^92^	Brazil	Cross - sectional	Obesity	20	48.54	0	100		Landmarks.				41.77	41.77		41.77						
Bruch, J, *et al.* ^93^	Brazil	Cross - sectional	Chronic Hepatitis C	58	51.6	44.8	55.2	Measuring tape	By the smallest circumference just above the laryngeal prominence with patient sitting down or standing up, with the spine erect and the head in the Frankfurt horizontal plane.		37	34	37.3	34.6	40	37.3						
Saldias, F, *et al.* ^94^	Chile	Cross - sectional	OSAHS	1464	54.4	65.23	34.77			40			41.6	43.2	38.2	41.6			79.6	26.6		
Oliveira, D, *et al.* ^95^	Brazil	Cross - sectional	Obesity	60	36	25	75	Measuring tape	Laryngeal prominence.				44.1			44.1						
Venturi, M, *et al.* ^96^	Brazil	Cross - sectional	Epilepsy	98	39.97	60.2	39.8				39	35	34.06	33.71	34.41	34.06						
Barbosa, L, *et al.* ^97^	Brazil	Cross - sectional	OSAHS	14	46.8	14.3	85.7	Measuring tape	Laryngeal prominence.		43	41	41.3			41.3						
Gallego, C, *et al.* ^98^	Argentina	Cross - sectional	OSAHS	22	61	86.37	13.63						44.5			44.5						
Serafim, P, *et al.* ^99^	Brazil	Cross - sectional	Obesity	120	XXX	24	76	Measuring tape					44.29	51.5	42	44.29						
Oriol, S, *et al.* ^100^	Mexico	Cross - sectional	Obesity	21	XXX	28.58	71.42		At the level of thyroid cartilage.				44.41	47.7	43.1	44.41						

Additionally, 12 studies ^53, 63, 70, 71, 78– 80, 87, 88, 93, 96, 97^ established different cut-off points for elevated NC for men and women, with a minimum and maximum value for men of 37cm and 43cm, respectively; while the values for women were 34cm and 41cm, respectively. The prevalence of high NC was available from 7 studies ^63, 70, 78, 86– 88, 90^, all of which used different thresholds for all, men and women. Overall, the cut-off points for high NC ranged between 40 cm and 42 cm; for men, the cut-off points ranged between 37 cm and 43 cm, while for women the range was between 34 cm and 41 cm ( Table 3). Based on these definitions, the prevalence of high NC in the general sample of patient population went from 30.4% to 86.6%; while for men and women it went from 34,2% to 95%, and from 26.6% to 65.8%, respectively ( Table 3). 

### Risk of bias of individual studies

From all the cross-sectional studies, only 4 studies ^19, 22, 25, 29^ are considered as a close representation of the general population. Finally, analyzing the risk of bias of the prevalence studies included, the majority represent a moderate risk ^17, 24, 29– 31, 33, 35– 43, 45, 47, 48, 50– 51, 53– 66, 68, 70– 73, 76– 84, 86, 89, 92– 100^ (57); some are low risk ^16, 18– 23, 25– 29, 32, 34, 44, 49^ (16); no study represents a high risk ( Table 4 – Table 6).

**Table 4.** Summary table about risk of bias for cross- sectional studies.

Risk of Bias – Prevalence Stuides											
First Author	External Validity				Internal Validity						11. Summary
	1. A close representation	2. True or close representation	3. Random selection	4. Non- response bias minimal	5. Directly from the subjects	6. Acceptable case definition	7. Measured was reliability and validity	8. Same mode of data collection	9. Length of the shortest prevalence period	10. Numerator(s) and denominator(s) appropate	
Moraes, W. *et al.* ^16^	NO	YES	YES	YES	YES	NA	UNCLEAR	YES	YES	YES	LOW
Neves, T. *et al.* ^17^	NO	UNCLEAR	UNCLEAR	NO	YES	NA	YES	YES	YES	YES	MODERATE
Ribeiro, L. *et al.* ^18^	NO	YES	YES	YES	YES	NA	YES	YES	YES	YES	LOW
Méndez, B. *et al.* ^19^	YES	YES	YES	YES	YES	NA	UNCLEAR	YES	YES	YES	LOW
Leite, J. *et al.* ^20^	NO	YES	YES	YES	YES	NA	UNCLEAR	YES	YES	YES	LOW
Tavares, C. *et al.* ^30^	NO	UNCLEAR	UNCLEAR	YES	YES	NA	UNCLEAR	YES	YES	YES	MODERATE
Dantas, E. *et al.* ^31^	NO	NO	NO	YES	YES	NA	YES	YES	YES	YES	MODERATE
De Siqueira, K. *et al.* ^32^	NO	YES	YES	YES	YES	NA	YES	YES	YES	YES	LOW
De Alexandria, F. *et al.* ^33^	NO	NO	NO	NO	YES	NA	YES	YES	YES	YES	MODERATE
Alves, H. *et al.* ^34^	NO	YES	YES	YES	YES	NA	YES	YES	YES	YES	LOW
Santos, D. *et al.* ^35^	NO	UNCLEAR	UNCLEAR	YES	YES	NA	UNCLEAR	YES	YES	YES	MODERATE
Pedreros, A. *et al.* ^36^	NO	UNCLEAR	UNCLEAR	YES	YES	NA	UNCLEAR	YES	YES	YES	MODERATE
Haueisen, M. *et al.* ^37^	NO	UNCLEAR	UNCLEAR	NO	YES	NA	YES	YES	YES	YES	MODERATE
Nogueira, M. *et al.* ^39^	NO	UNCLEAR	UNCLEAR	YES	YES	NA	YES	YES	YES	YES	MODERATE
Barbosa, P. *et al.* ^40^	NO	UNCLEAR	UNCLEAR	YES	YES	NA	YES	YES	YES	YES	MODERATE
Frizon, V. *et al.* ^41^	NO	UNCLEAR	UNCLEAR	YES	YES	NA	YES	YES	YES	YES	MODERATE
Coelho, H. *et al.* ^42^	NO	NO	NO	NO	YES	NA	YES	YES	YES	YES	MODERATE
Da Silva, A. *et al.* ^43^	NO	UNCLEAR	UNCLEAR	YES	YES	NA	YES	YES	YES	YES	MODERATE
Zonato, A. *et al.* ^53^	NO	UNCLEAR	UNCLEAR	YES	YES	NA	UNCLEAR	YES	YES	YES	MODERATE
Sutherland, K. *et al.* ^54^	NO	UNCLEAR	UNCLEAR	YES	YES	NA	YES	YES	YES	YES	MODERATE
Pinto, J. *et al.* ^55^	NO	UNCLEAR	UNCLEAR	NO	YES	NA	UNCLEAR	YES	YES	YES	MODERATE
Oliveira, N. *et al.* ^56^	NO	NO	NO	YES	YES	NA	UNCLEAR	YES	YES	YES	MODERATE
Musman, S. *et al.* ^57^	NO	UNCLEAR	UNCLEAR	YES	YES	NA	YES	YES	YES	YES	MODERATE
Salas, C. *et al.* ^58^	INO	UNCLEAR	UNCLEAR	YES	YES	NA	UNCLEAR	YES	YES	YES	MODERATE
Saban, M. *et al.* ^59^	NO	UNCLEAR	UNCLEAR	NO	YES	NA	UNCLEAR	YES	YES	YES	MODERATE
Souza, F. *et al.* ^60^	NO	UNCLEAR	UNCLEAR	YES	YES	NA	UNCLEAR	YES	YES	YES	MODERATE
Boemeke, L. *et al.* ^63^	NO	UNCLEAR	UNCLEAR	YES	YES	NA	YES	YES	YES	YES	MODERATE
Hiray, M. *et al.* ^64^	NO	UNCLEAR	UNCLEAR	YES	YES	NA	UNCLEAR	YES	YES	YES	MODERATE
Borges, P. *et al.* ^65^	NO	UNCLEAR	UNCLEAR	YES	YES	NA	UNCLEAR	YES	YES	YES	MODERATE
Saldaña, R. *et al.* ^66^	NO	UNCLEAR	UNCLEAR	YES	YES	NA	UNCLEAR	YES	YES	YES	MODERATE
Jorquera, A. *et al.* ^68^	NO	UNCLEAR	UNCLEAR	YES	YES	NA	UNCLEAR	YES	YES	YES	MODERATE
Rodrigues, A. *et al.* ^70^	NO	NO	NO	YES	YES	NA	YES	YES	YES	YES	MODERATE
Aguiar, I. *et al.* ^71^	NO	UNCLEAR	UNCLEAR	YES	YES	NA	UNCLEAR	YES	YES	YES	MODERATE
Saldías, P. *et al.* ^87^	NO	UNCLEAR	UNCLEAR	YES	YES	NA	UNCLEAR	YES	YES	YES	MODERATE
Chávez, C. *et al.* ^72^	NO	UNCLEAR	UNCLEAR	YES	YES	NA	UNCLEAR	YES	YES	YES	MODERATE
De Menezes, R. *et al.* ^73^	NO	UNCLEAR	UNCLEAR	NO	YES	NA	UNCLEAR	YES	YES	YES	MODERATE
Faria, N. *et al.* ^76^	NO	UNCLEAR	UNCLEAR	YES	YES	NA	UNCLEAR	YES	YES	YES	MODERATE
Schommer, V. *et al.* ^77^	NO	UNCLEAR	UNCLEAR	YES	YES	NA	YES	YES	YES	YES	MODERATE
Amaro, T. *et al.* ^78^	NO	UNCLEAR	UNCLEAR	YES	YES	NA	UNCLEAR	YES	YES	YES	MODERATE
Volaco, A. *et al.* ^21^	NO	NO	YES	YES	YES	NA	YES	YES	YES	YES	LOW
Stabe, C. *et al.* ^22^	YES	YES	NO	NO	YES	NA	YES	YES	YES	YES	LOW
Zanuncio, V. *et al.* ^23^	NO	NO	YES	YES	YES	NA	YES	YES	YES	YES	LOW
Chaves, T. *et al.* ^24^	NO	NO	YES	NO	YES	NA	NO	YES	YES	YES	MODERATE
Alfie, J. *et al.* ^25^	YES	YES	YES	YES	YES	NA	YES	YES	YES	YES	LOW
Soares, M. *et al.* ^26^	NO	YES	YES	YES	YES	NA	NO	YES	YES	YES	LOW
Ruiz, A. *et al.* ^27^	NO	YES	YES	YES	YES	NA	YES	YES	YES	YES	LOW
Polesel, D. *et al.* ^28^	NO	YES	YES	YES	YES	NA	NO	YES	YES	YES	LOW
Mora, R. *et al.* ^29^	YES	YES	YES	YES	YES	NA	YES	YES	YES	YES	LOW
Closs, V. *et al.* ^44^	NO	YES	UNCLEAR	YES	YES	NA	NO	YES	YES	YES	LOW
Petreça, D. *et al.* ^45^	NO	NO	NO	NO	YES	NA	YES	YES	YES	YES	MODERATE
Peralta, C. *et al.* ^47^	NO	YES	UNCLEAR	NO	YES	NA	NO	YES	YES	YES	MODERATE
Dos reis, E. *et al.* ^48^	NO	YES	UNCLEAR	NO	YES	NA	NO	YES	YES	YES	MODERATE
Ferreira, A. *et al.* ^49^	NO	YES	YES	YES	YES	NA	YES	YES	YES	YES	LOW
Ramires, A. *et al.* ^45^	NO	UNCLEAR	UNCLEAR	YES	YES	NA	YES	YES	YES	YES	MODERATE
Mora, R. *et al.* ^29^	NO	UNCLEAR	UNCLEAR	YES	YES	NA	YES	YES	YES	YES	MODERATE
Sgariboldi, D. *et al.* ^51^	NO	UNCLEAR	UNCLEAR	YES	YES	NA	YES	YES	YES	YES	MODERATE
Nerbass, F. *et al.* ^79^	NO	UNCLEAR	UNCLEAR	YES	YES	NA	NO	YES	YES	YES	MODERATE
Lucas, E. *et al.* ^80^	NO	UNCLEAR	UNCLEAR	YES	YES	NA	YES	YES	YES	YES	MODERATE
Freire, L. *et al.* ^81^	NO	UNCLEAR	UNCLEAR	YES	YES	NA	NO	YES	YES	YES	MODERATE
Sgariboldi, D. *et al.* ^82^	NO	UNCLEAR	UNCLEAR	YES	YES	NA	YES	YES	YES	YES	MODERATE
Martinho, F. *et al.* ^83^	NO	UNCLEAR	UNCLEAR	NO	YES	NA	NO	YES	YES	YES	MODERATE
Correa, M, *et al.* ^84^	NO	UNCLEAR	UNCLEAR	YES	YES	NA	YES	YES	YES	YES	MODERATE
Menezes, D, *et al.* ^86^	NO	UNCLEAR	UNCLEAR	YES	YES	NA	YES	YES	YES	YES	MODERATE
Miño, F, *et al.* ^89^	NO	UNCLEAR	UNCLEAR	YES	YES	NA	NO	YES	YES	YES	MODERATE
Padilha, L, *et al.* ^92^	NO	UNCLEAR	UNCLEAR	YES	YES	NA	NO	YES	YES	YES	MODERATE
Bruch, J, *et al.* ^93^	NO	UNCLEAR	UNCLEAR	YES	YES	NA	YES	YES	YES	YES	MODERATE
Saldias, F, *et al.* ^94^	NO	UNCLEAR	UNCLEAR	YES	YES	NA	NO	YES	YES	YES	MODERATE
Oliveira, D, *et al.* ^95^	NO	UNCLEAR	UNCLEAR	YES	YES	NA	YES	YES	YES	YES	MODERATE
Venturi, M, *et al.* ^96^	NO	UNCLEAR	YES	YES	YES	NA	NO	YES	YES	YES	MODERATE
Barbosa, L, *et al.* ^97^	NO	UNCLEAR	UNCLEAR	YES	YES	NA	YES	YES	YES	YES	MODERATE
Gallego, C, *et al.* ^98^	NO	UNCLEAR	UNCLEAR	YES	YES	NA	NO	YES	YES	YES	MODERATE
Serafim, P, *et al.* ^99^	NO	UNCLEAR	UNCLEAR	NO	YES	NA	NO	YES	YES	YES	MODERATE
Oriol, S, *et al.* ^100^	NO	UNCLEAR	UNCLEAR	YES	YES	NA	NO	YES	YES	YES	MODERATE

**Table 5.** Summary table about risk of bias for case - control studies.

Risk of Bias – Case & Control Studies								
First Author	Selection				Comparability	Exposure		
	1. Is the case definition adequate?	2. Representativeness of the cases	3. Selection of Controls	4. Definition of Controls		5. Ascertainment of exposure	6. Same method of ascertainment for cases and controls	7. Non- Response rate
Genta, P. *et al.* ^38^	B	A	B	B	NA	NA	A	A
De Paiva, R, *et al.* ^52^	C	A	B	A	NA	NA	A	A
De Castro, J, *et al.* ^62^	A	A	B	B	NA	NA	A	A
Castorena- Maldonado, A, *et al.* ^67^	A	A	B	A	NA	NA	A	A
Cunha, F, *et al.* ^74^	A	A	B	A	NA	NA	A	A
Magalhaes, E, *et al.* ^85^	A	A	B	A	NA	NA	A	A
Saldias, P, *et al.* ^87^	A	A	C	B	NA	NA	A	A

**Table 6.** Summary table about risk of bias for cohort studies.

Risk of Bias – Cohort Studies								
First Author	Selection				Comparability	Exposure		
	1. Representativeness of the exposed cohort	2. Selection of the non- exposed cohort	3. Ascertainment of exposure	4. Demonstration that outcome of interest was not present at start of study		5. Assessment of outcome	6. Was follow- up long enough for outcomes to occur	7. Adequacy of follow up of cohorts
Moura, P, *et al.* ^60^	B	C	A	A	NA	NA	B	A
De Castro, J, *et al.* ^69^	B	A	A	B	NA	NA	UNCLEAR	A
Prescinotto, R, *et al.* ^75^	B	A	A	A	NA	NA	B	A
Pizarro-Montaner, C. *et al.* ^46^	C	A	A	B	NA	Unclear	A	A
Mendes, C, *et al.* ^88^	C	A	A	A	NA	Unclear	A	A
Garcia, J, *et al.* ^90^	C	A	A	B	NA	Unclear	A	A
Lima, J, *et al.* ^91^	C	A	A	A	NA	Unclear	A	A

Regarding the risk of bias of the case-control studies, in all the studies (7) ^38, 52, 62, 67, 74, 85, 87^ the sample selection adequately represented the corresponding cases. In addition, concerning the risk of bias of the cohort studies (7) ^46, 60, 69, 75, 88, 90, 91^, all the studies had an adequate follow-up of the cohorts ( Table 4 – Table 6). 

## Discussion

### Summary of evidence

This is a systematic review and meta-analysis to estimate the mean NC and the prevalence of high NC in adults from LAC. We summarized evidence from 14 studies in the general population; 23 from captive populations (e.g., students); and 49 studies with patients (mostly OSAHS and obesity). The mean NC in the general population ranged between 33.60cm and 36.98cm ^22, 24^, and the prevalence of elevated NC ranged between 37.00% and 57.69% ^18, 25^. The average NC in captive populations went from 33.00cm to 42.00cm ^33, 38^, and the prevalence of elevated NC varied between 54.25% and 62.00% ^42, 48^. In the patients-based studies, the minimum mean NC was 33.41cm whilst the maximum was 44.50cm ^70, 98^; the prevalence of high NC ranged between 30.40% and 86.60% ^63, 70^. NC raises as a relevant anthropometric indicator which could complement information based on BMI and WC for the early identification of cardio-metabolic and other diseases in LAC. This systematic review provides the first regional overview of mean NC and prevalence of high NC in LAC.

### Limitations of the reviewed reports

The main limitation we found in the original reports was the lack of details on how NC was measured; that is, they did not consistently report the instruments (e.g., inelastic tape) and how NC was assessed. The same problem was observed regarding the cut-off points to define high NC; that is, there were not consistent and comparable thresholds. These limitations have overall implications and for our review as well. First, the high heterogeneity in methods and definitions hampers comparisons across studies/countries; also, the heterogeneity makes it difficult to study time trends. Regarding our review, the inconsistent methods and lack of standard definitions could explain the large heterogeneity reported in the meta-analyses, and also prevented us from conducting more meta-analyses, for example of prevalence estimates. We argue that the dearth of homogenous reporting and methodology is due to the lack of international standardization in the measurement of NC, which could be explained by how novel this anthropometric indicator is. There is a need for an international standardized measurement of NC which would allow cross-country and time trends analyses. 

Another limitation of the reviewed studies was that only 4 ^19, 22, 25, 29^ were conducted with a nationally representative sample. Therefore, information on mean NC and prevalence of high NC at the national level is missing in most countries of LAC. NC is an inexpensive and non-invasive anthropometric indicator, as it is the case with BMI or WC. Once standard procedures to measure NC and standard thresholds to define high NC are defined, NC could be implemented in large national surveys (e.g., DHS or WHO STEPS) to expand the arsenal of anthropometric indicators strongly associated with morbidity and mortality of cardio-metabolic diseases ^7– 10^. 

### Limitations of the review

Our review has some limitations. First, although we used major global search engines (MEDLINE, EMBASE and Global Health), and one specific for LAC (LILACS), we did not search grey literature sources. These sources could have contributed few more results to our review; however, we doubt they would have substantially changed the conclusions. Most likely, they would have exhibited the same -or more severe- limitations as those herein pinpointed. Second, some studies did not report all the information. Even though we tried to contact the authors of the reports with missing information, 6/16 answered to our requests. As NC becomes a more popular anthropometric indicator, and standard methods and definitions are established by international or regional organizations, we believe that studies including NC information would provide more comprehensive methods and results. Hopefully, our work would spark interest in NC and about the relevance to have standard procedures, as there are with BMI and other anthropometric indicators. Third, our review could not find estimates for all countries in LAC, and neither did other multi-country endeavors (e.g., ELANS) ^101^. Therefore, we cannot conclusively state that our estimates represent the scenario across the region. Nonetheless, our work adds to the regional literature with a summary from captive populations and patients-based studies; also, we provide a risk of bias assessment and discuss the limitations of original reports and propose recommendations. 

### Results in context

NC has been associated with several cardiometabolic risk factors: insulin resistance, elevated cholesterol, triglycerides, LDL-cholesterol and obesity ^102, 103^. Moreover, NC has also been associated with SAHOS ^104^. Nevertheless, and despite that NC appears to be as good (or even better) as other anthropometric indicators (e.g., BMI), NC has not been subject to extensive research. In this work we propose the first systematic review and meta-analysis to reveal the overall mean NC in LAC, and to highlight research needs. Our work is the starting point to raise awareness about NC as a potential anthropometric indicator, while signaling the need for NC cut-off points in LAC.

A multinational study (ELANS conducted in 2014–2015) ^101^ was conducted in eight LAC countries and they found a mean NC of 35.60cm, which is virtually the same as our pooled mean estimate. This similarity could be explained by the fact that we covered the same countries. Notably, the ELANS study included populations in more countries than those herein summarized, yet we included older and more recent studies, and we also summarized evidence from a larger sample. Overall, mean NC in LAC appears to be ~35cm, though this deserves further verification following consistent methods and including countries for which evidence is still unavailable. 

Studies in Asia reported a mean NC between 31cm and 44cm ^102, 103^. Our pooled estimates fall within this range. As it is the case with other anthropometric indicators (e.g., BMI), LAC is usually in the middle of the distribution ^105^. Reasons behind this could be diet and nutrition, phenotypes, opportunities to exercise, and access to preventive healthcare, all of which vary widely across countries and regions. As more evidence about NC in LAC becomes available, we would be in a stronger position to study determinants and outcomes for high NC to identify reasons for cross-country and cross-region comparisons.

The mean NC and prevalence of high NC was larger in captive populations in comparison to the general population. This could be explained by the underlying profile of each captive group. For example, in bus drives, miners, sedentary women and adults - elderly waiting for medical attention, those variables were higher due to the fact that these people have a long working day which could condition a sedentary lifestyle. However, in other population groups (e.g., university students, health professionals, outdoor gym users) the mean NC and prevalence of high NC was lower than in the general population. This could be because these groups have healthier lifestyles and are more concerned about their health (due to their profession).

We also found that the mean NC in the group of OSAH and obese is higher than the general population (41.09cm and 42.56cm vs 35.69cm). This is concordant with the studies that considered NC as an anthropometric measure useful for assessing the risk and severity of OSAH and also it is known for its strong relationship with obesity ^106, 107^. A higher NC in this group of patients can be explained by the accumulation of fat around the neck contributing to the airway narrowing and at the same time facilitating its obstruction ^108, 109^. NC could be incorporated as part of the standard of care for OSAH patients.

Currently, there are no guides that include NC as an official anthropometric measure; however, there are studies that found NC as a reliable index and highlight the fact that it is an economical test easy to use which takes less time and correlates well with other anthropometric parameters such as BMI, WC and hip circumference ^110, 111^. Our findings indicate that NC could be used either in clinical practice and epidemiologic studies.

## Conclusions

In this systematic review and meta-analysis, the mean NC in LAC was 35cm in the general population; although there were different thresholds, the prevalence of high NC ranged between 37.00% and 57.69%. The methodology to measure NC was inconsistently reported and evidence lacks from several countries in LAC. Even though NC could be a novel anthropometric indicator closely related with different diseases and health outcomes, NC has been seriously understudied in LAC. This work highlights the current evidence about NC in LAC and pinpoints research gaps.

## Data availability

### Underlying data

All data underlying the results are available as part of the article and no additional source data are required.

### Extended data

Figshare: Supplementary Material. https://doi.org/10.6084/m9.figshare.13550534 ^13^

This project contains the following extended data:

- Supplementary Material.docx (Document with study search strategy)

### Reporting guidelines

Figshare: PRISMA checklist for ‘Neck circumference in Latin America and the Caribbean: A systematic review and meta-analysis’ https://doi.org/10.6084/m9.figshare.13550534 ^13^
