# Supplementary material for: Neck circumference in Latin America and the Caribbean: A systematic review and meta-analysis
Source: Wellcome Open Res. 2021 Jan 26;6:13. [Version 1] doi: 10.12688/wellcomeopenres.16560.1 (PMC8080980; doi:10.12688/wellcomeopenres.16560.1)
Supplement: Peer review history

# Peer review: Reviewer response for version 1

**DOI:** 10.21956/wellcomeopenres.18249.r43403

**Published:** 2021-04-22

**Authors:** Mary Penny

**Affiliations:** 1 Instituto de Investigación Nutricional, Lima, Peru

This is an interesting article that reviews the little-used measurement of neck circumference and the advantages and limitations of this measure as reported in the literature. The review of the use in different studies is helpful and the authors suggest that it would be a convenient and useful addition to the arsenal of anthropometric measures currently in use.

 The review and suggestion for the use of measuring neck circumference is convincing as the neck is relatively accessible for measurement and this could be a useful addition in studies that require anthropometry.

 I recommend indexing.

Are the rationale for, and objectives of, the Systematic Review clearly stated?

Yes

Is the statistical analysis and its interpretation appropriate?

Yes

Are sufficient details of the methods and analysis provided to allow replication by others?

Yes

Are the conclusions drawn adequately supported by the results presented in the review?

Yes

Reviewer Expertise:

Population research which usually includes anthropometric assessment of candidates for randomized controlled studies

I confirm that I have read this submission and believe that I have an appropriate level of expertise to confirm that it is of an acceptable scientific standard.

# Peer review: Reviewer response for version 1

**DOI:** 10.21956/wellcomeopenres.18249.r42342

**Published:** 2021-02-02

**Authors:** Jose Alfie

**Affiliations:** 1 Hypertension Section, Internal Medicine Department, Hospital Italiano de Buenos Aires, Buenos Aires, Argentina; 2 Sociedad Argentina de Hipertension Arterial, Buenos Aires, Argentina

The increase in neck circumference, a measure of ectopic fat, is associated with higher cardiometabolic risk and obstructive sleep apnea beyond classical anthropometric measures of obesity.

 The authors performed a systematic review and meta-analysis to estimate the mean neck circumference and the prevalence of high neck circumference in Latin America and the Caribbean.

 I find the article correctly written and analyzed.

 Although the measurement of neck circumference differs between studies, the net impact of these differences is probably minimal compared to different definitions of abdominal obesity. This, added to the fact that to measure the circumference of the neck it is not necessary to ask the patient to get up from the chair or to remove their clothes, it represents advantages over other anthropometric measurements.

 The objective of the study was to establish mean values and the prevalence of high neck circumference in Latin America and the Caribbean. This was based on statistical definitions provided by the selected studies. Another approach to define normality and cutoff values could be based on the consequences of increased neck circumference on metabolism, blood pressure, and obstructive sleep apnea.

Are the rationale for, and objectives of, the Systematic Review clearly stated?

Yes

Is the statistical analysis and its interpretation appropriate?

Yes

Are sufficient details of the methods and analysis provided to allow replication by others?

Yes

Are the conclusions drawn adequately supported by the results presented in the review?

Yes

Reviewer Expertise:

My area of expertise is high blood pressure. I am the first author of one of the articles selected in the current metaanalysis (25).

I confirm that I have read this submission and believe that I have an appropriate level of expertise to confirm that it is of an acceptable scientific standard.
